# Influence of Seasonal Food Availability on the Dynamics of Seabird Feeding Flocks at a Coastal Upwelling Area

**DOI:** 10.1371/journal.pone.0131327

**Published:** 2015-06-30

**Authors:** Cristóbal Anguita, Alejandro Simeone

**Affiliations:** Departamento de Ecología y Biodiversidad, Facultad de Ecología y Recursos Naturales, Universidad Andrés Bello, Santiago, Chile; University of Vigo, SPAIN

## Abstract

The formation of multi-species feeding flocks (MSFFs) through visual recruitment is considered an important strategy for obtaining food in seabirds and its functionality has been ascribed to enhanced foraging efficiency. Its use has been demonstrated in much of the world's oceans and includes numerous species. However, there is scant information on the temporal stability of the composition and abundance of MSFFs as well as the effect of seasonal food availability on their dynamics. Between July 2006 and September 2014, we conducted monthly at-sea seabird counts at Valparaiso Bay (32°56′ to 33°01′S, 71°36′ to 71°46′W) within the area of influence of the Humboldt Current in central Chile. This area is characterized by a marked seasonality in primary and secondary production associated with upwelling, mainly during austral spring-summer. Based on studies that provide evidence that flocking is most frequent when food is both scarce and patchy, we hypothesized that seabird MSFF attributes (i.e. frequency of occurrence, abundance and composition) will be modified according to the seasonal availability of food. Using generalized linear models (GLMs), our results show that the contrasting seasonality in food availability of the study area (using chlorophyll-*a* concentration as a proxy) had no significant influence on MSFF attributes, sparsely explaining their variations (P>0.05). Rather than seasonal food availability, the observed pattern for MSFF attributes at Valparaiso Bay suggests a substantial influence of reproductive and migratory (boreal and austral migrants) habits of birds that modulates MSFF dynamics consistently throughout the whole year in this highly variable and patchy environment. We highlight the importance of visual recruitment as a mechanism by which migratory and resident birds interact. This would allow them to reduce resource unpredictability, which in turn has a major impact on structuring seabird’s MSFF dynamics.

## Introduction

To obtain information on certain quality traits of the environment, animals usually rely on the presence or behavior of other individuals [1–3]. A widely used feeding strategy by birds is the formation of multi-species feeding flocks (MSFFs). The functionality of MSFFs has been traditionally attributed to reducing the risk of predation and/or increase foraging efficiency [4–6]. Seabirds are predators that usually do not face high risk of predation at sea [7], so MSFFs function has been associated mostly to foraging efficiency [8]. A major mechanism used by seabirds to find their prey is to observe the behavior of other birds, a strategy called local enhancement [8–12]. This mechanism assumes that it is easier to detect other birds feeding than directly detecting prey (in the case of seabirds, usually fish, crustaceans and cephalopods [13]). Recently, Thiebault et al. [14] showed for the Cape gannet (*Morus capensis*), depending on the size of the aggregations, that this mechanism is effective up to 40 km. Detectability of prey is also determined by the species role and may include initiators or catalysts (e.g. gulls), joiners (e.g. shearwaters), divers (e.g. cormorants) and kleptoparasites (e.g. jaegers [8,15]).

Formation of MSFFs in seabirds has been observed in much of the world's oceans and may sometimes exceed one million birds [8,16–26]. In the eastern tropical Pacific, Ballance et al. [20] identified three types of MSFFs depending on surface water productivity, which directly affected energetic costs of flight and interference competition. Silverman and Veit [24] observed that the abundance and composition of MSFFs differed dramatically between two oceanographic regions in South Georgia. Differences observed by these authors were attributed to the vertical availability of krill (*Euphausia superba*) [24]. In the Humboldt Current System (HCS), two studies have shown that MSFFs are important feeding strategies for seabirds. In northern Peru, Duffy [16] found (during spring and summer) a high frequency of occurrence of guano birds, where 99% of the individuals were seen foraging exclusively in MSFFs. In addition, he noted substantial compositional changes from guano birds MSFFs formed on shoals of anchovy (*Engraulis ringens*) to MSFFs composed mainly of gulls and terns over zooplankton patches [16]. In northern Chile, Weichler et al. [25] found that during summer, the occurrence of MSFFs was the most influential variable of the distributional patterns of endemic seabirds of the HCS and these were composed mainly of Gray gull, Humboldt penguin, Peruvian booby and Guanay cormorant (for scientific names, see Table 1).

**Table 1:** Seasonal variation in species abundance (number of individuals observed in MSFFs per month) and its relation with primary production.

			Seasons		Chl-*a*			Seasons x Chl-*a*	
Common name	Scientific name	Code	Dev.	Pr(>Dev)	Dev.	Pr(>Dev)	Coef.	Dev.	Pr(>Dev)
Southern fulmar	*Fulmarus glacialoides*	SOFU	23.92	**<0.001**	9.43	**0.002**	**-1.37**	1.41	0.796
Franklin's gull	*Leucophaeus pipixcan*	FRGU	20.73	**<0.001**	0.03	0.787	-0.12	2.14	0.463
Pink-footed shearwater	*Ardenna creatopus*	PISH	18.39	**0.012**	0.00	0.890	1.26	1.04	0.538
Cape petrel	*Daption capense*	CAPE	17.12	**0.002**	0.32	0.250	-0.32	0.14	1.000
Gray gull	*Leucophaeus modestus*	GRGU	15.99	**0.003**	0.52	0.362	-0.20	4.91	0.148
White-chinned petrel	*Procellaria aequinoctialis*	WHPE	15.99	**0.014**	11.15	**0.001**	**-1.02**	4.01	0.328
Magellanic penguin	*Spheniscus magellanicus*	MAPE	12.03	**0.017**	2.38	0.121	-0.40	4.58	0.305
Sooty shearwater	*Ardenna grisea*	SOSH	11.66	**0.027**	1.18	0.312	-0.35	4.94	0.334
Red phalarope	*Phalaropus fulicarius*	REPH	10.36	**0.013**	0.91	0.076	1.80	0.26	0.917
Brown-hooded gull	*Chroicocephalus maculipennis*	BRGU	9.16	**0.046**	4.62	**0.038**	**-0.96**	3.56	0.328
Black-browed albatross	*Thalassarche melanophris*	BBAL	8.85	0.066	0.04	0.832	-0.11	9.81	0.230
South American tern	*Sterna hirundinacea*	SOTE	8.17	0.110	0.08	0.722	1.16	0.97	0.632
Arctic tern	*Sterna paradisaea*	ARTE	7.92	0.098	0.43	0.457	0.21	2.96	0.423
Neotropic cormorant	*Phalacrocorax brasilianus*	NECO	7.88	0.068	0.19	0.659	-0.04	1.69	0.499
Inca tern	*Larosterna inca*	INTE	7.69	**0.033**	0.09	0.791	-0.04	2.68	0.571
Peruvian booby	*Sula variegata*	PEBO	6.97	0.145	3.24	0.146	0.40	0.99	0.878
Kelp gull	*Larus dominicanus*	KEGU	6.72	0.120	2.02	0.239	-0.29	2.88	0.611
Peruvian diving-petrel	*Pelecanoides garnotii*	DIPE	6.58	0.249	3.21	0.206	-0.86	1.68	0.797
Parasitic jaeger	*Stercorarius parasiticus*	PAJA	5.20	0.300	0.05	0.793	0.09	1.27	0.580
Westland petrel	*Procellaria westlandica*	WEPE	5.13	0.191	3.41	**0.026**	**-8.64**	0.38	0.862
Undetermined penguin	*Spheniscus* spp. juvenile	SPHE	4.52	0.537	0.08	0.746	0.72	1.34	0.774
Guanay cormorant	*Phalacrocorax bougainvillii*	GUCO	3.98	0.422	0.56	0.571	-0.23	2.53	0.672
Wilson's storm-petrel	*Oceanites oceanicus*	WIST	3.00	0.613	0.50	0.387	0.23	2.11	0.613
Peruvian pelican	*Pelacanus thagus*	PEPE	2.82	0.416	0.88	0.509	-0.13	4.11	0.488
Chilean skua	*Stercorarius chilensis*	CHSK	2.63	0.726	0.33	0.575	0.41	1.15	0.764
Salvin's albatross	*Thalassarche salvini*	SAAL	1.93	0.699	0.19	0.673	-0.37	5.96	0.061
Red-legged cormorant	*Phalacrocorax gaimardi*	RECO	1.64	0.792	0.93	0.323	-0.56	3.67	0.436
Humboldt penguin	*Spheniscus humboldti*	HUPE	0.91	0.942	0.90	0.430	0.16	3.78	0.421

Species ordered from largest to smallest explained deviance on seasonal variation (GLMs, negative binomial error distribution). Coefficients (Coef.) were estimated considering only the productivity as a predictor. Chl-*a*: chlorophyll-*a* concentration; Dev: Deviance; x: indicates interaction between predictive variables.

While Ballance et al. [20] and Silverman and Veit [24] identify the influence of spatial variation in food availability on composition and abundance of MSFFs, it has not been clearly demonstrated the effect of temporal food availability on MSFF attributes. At the HCS, both studies mentioned above [16,25] were performed over a single season in spring and summer, a period characterized by high food availability due to upwelling affecting these coastal areas of the Southeast Pacific. This situation hinders the understanding of whether the observed patterns are stable over the time or are subject to seasonal fluctuations. In land birds, studies in different habitats and seasons have shown that the vast majority of birds adhere to MSFFs when there is low availability of resources or when they are scattered, which is consistent with the idea that this strategy optimizes foraging [27–33].

The coast of Valparaiso in central Chile is characterized by a marked seasonality in primary production due to the combined effects of surface irradiation and fertilization by nutrient-rich upwelled waters [34–37]. In this area, primary production is highest during austral spring-summer seasons which in turn produce and concentrate high abundance of zooplankton and fishes [38–42], the main prey for seabirds [13]. During winter, primary production decreases as well as food supply for birds [38–42]. This situation, coupled with the high richness and abundance of endemic, resident and migratory seabirds at central Chile [43–46], provides an interesting scenario to test the effects of seasonal food availability on MSFF attributes. We hypothesized that MSFF frequency of occurrence, abundance and composition are modified according to the seasonal availability of food. We predict that in seasons with lower food availability (autumn-winter), MSFFs will be more frequent and abundant, while in seasons with higher food availability (spring-summer), MSFFs will be less frequent and will contain fewer individuals.

## Methods

### Data collection

**Fig 1:**
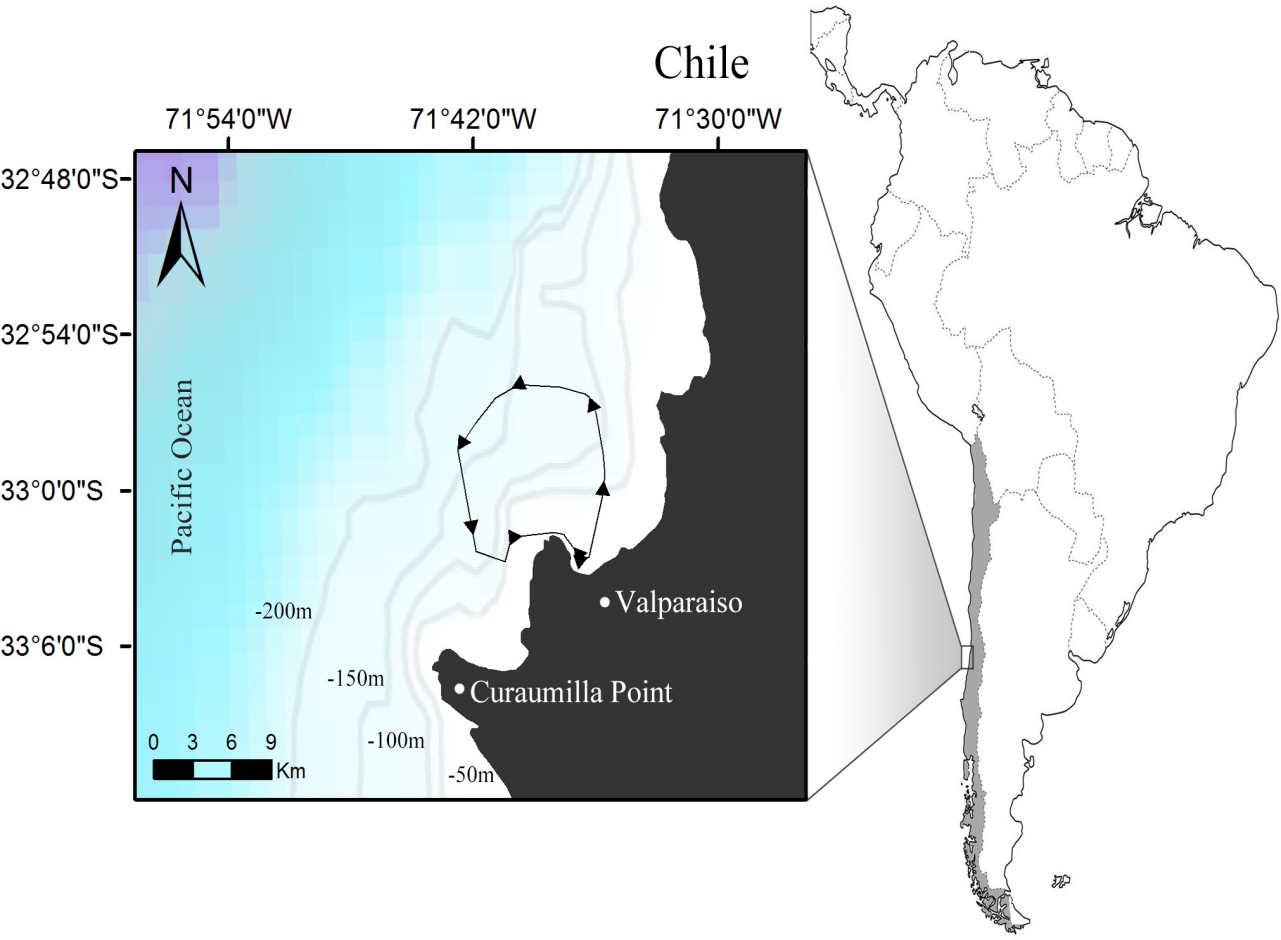
Study area in the Valparaiso Bay, central Chile. Minimum convex polygon (110 km^2^) shows 95% of GPS positions obtained during the period of study (2006–2014). The polygon depicts transect and direction regularly followed during the study. The bathymetry of the study area with the first four isobaths within the continental shelf is shown.

No animal research or field permits were required to perform this study. From July 2006 throughout September 2014, we performed monthly counts of seabirds within the Valparaiso Bay (32°56′ to 33°01′S, 71°36′ to 71°46′W) in central Chile, encompassing a maximum area of *ca* 110 km^2^ (Fig 1). The counts were performed using 10×42 binoculars from a 10m long motor vessel (120 HP) usually between 1030 and 1330 hours (UTM/GTM-3 hours) using a standard method for counting seabirds at sea [47–51]. In this method, the birds are counted by two independent teams of two observers at each side of the boat along a line transect. Data recorded by each group of observers included the identity of the species engaged in MSFFs, the behavior (using a standardized coding after [51]), the abundance and the distance to the boat (Table 1). Birds observed in MSFFs were counted ‘in transect’ if they were within a 250m-wide distance band perpendicular to the boat. This band was subdivided into three discrete bands: 0 to 50 (A), 50 to 100 (B), and 100 to 250 m (C). The birds outside this area were considered as to be ‘outside transect’ and were not considered in the data analysis. The names and systematics followed Remsen et al. [52].

Transects extended up to 15 km offshore within the limits of the continental shelf (Fig 1), and the navigation speed was kept at constant as possible between 10 and 12 km/h, so as to obtain adequate detection of birds [53]. As much as weather and wave conditions made possible, each month, we kept the same track and trip duration (mean ± SD: 3 ± 0.3 h, n = 99). The counts were performed only if a minimal set of weather and sea conditions were present, including visibility >1 km and sea state ≤5 (Beaufort scale). The position and speed of the boat were recorded every 10 min using a GPS.

The methodology described above was applied only to those birds that were detected as part of feeding groups, excluding those groups of birds involved in activities other than feeding (e.g. resting, preening and traveling). For the purposes of this study, we considered an MSFF to be any group of two or more birds of different species observed feeding in the same event (patch) at a fine spatial scale (meters to hundreds of meters, see [54]), which depended on the species involved and the ‘nature’ of the prey which triggered MSFF formation (e.g. zooplankton *vs* shoals). MSFF attributes considered in this study were: (1) frequency of occurrence: number of MSFFs observed per month, considering all sampling months during the study period; (2) abundance: total number of individuals observed in MSFFs per month, considering all species and all sampling months during the study period and (3) composition: total number of individuals per species observed in MSFFs per month, considering only sampling months that MSFFs were recorded.

### Oceanographic data

We used remotely sensed at-sea primary production (chlorophyll*-a* concentration) as a proxy for food availability in our study. We used chlorophyll-*a* because: (1) it is available in the same spatial and temporal scale as our bird counts, (2) seasonality in primary and secondary production in the study area is strong (which is well represented by remote-sensed data used in this study, see Results) and (3) there is a strong synchronization (i.e. short time lag) between phyto-zooplankton production and planktivorous fish abundance documented for south-central Chile (see Discussion). Although it would have been desirable to use direct data on zooplankton and/or forage fish to relate with MSFF attributes, such information is not available at the temporal (season) and spatial (Valparaiso Bay) scales requested by our analysis. The use of chlorophyll-*a* concentration as a proxy for food availability, however, has been previously utilized for similar purposes in other upwelling regions; particularly Benguela and California [55–59] with satisfactory results (see discussion).

Monthly mean concentration of chlorophyll*-a* (mg/m^3^) was obtained using the average of the study area (considering Curaumilla Point (33°06′S, 71°43′W) due to the oceanographic influence of this area on the coast of Valparaiso [34,35,37,60])**.** Moderate-resolution imaging spectroradiometer (MODIS-aqua) with a resolution of 4 km (whose frequency measurement are within 1–2 days), were downloaded from the Giovanni online data system (http://giovanni.gsfc.nasa.gov).

For MSFF attributes and for oceanographic data, the sampling months were considered as replicates and were grouped within their corresponding austral seasons (autumn: March-April-May; winter: June-July-August; spring: September-October-November; summer: December-January-February). During the study period there have been no significant anomalies in the Oceanic Niño Index at the Pacific Ocean (http://www.cpc.noaa.gov). Indeed, primary production at the coast of Valparaiso showed non-significant interannual (2006–2014) variation (Kruskal-Wallis *X* ^2^ = 4.55; P = 0.839).

### Data Analysis

#### Primary production of the study area

Due to the lack of normality of the data, the Kruskal-Wallis test followed by the Mann- Whitney *U* test (as a *post-hoc*) was used to analyze seasonal variation of primary production (monthly average) in the study area.

To examine the spatiotemporal variation in primary production between the most contrasting seasons of the study area (winter and spring), we computed and plotted the average monthly concentration of chlorophyll*-a* during the period of study, using the *raster* package [61] in R (http://CRAN.R-project.org).

#### Frequency of occurrence and abundance of MSFFs

In order to determine the seasonal variation in MSFF attributes and relate their variations to food availability, we use generalized linear models (GLMs) with a negative binomial error distribution (appropriate in cases of overdispersion [62,63]) where seasonal attributes were the dependent variables and seasonality with primary production were the predictive and interactive variables. GLMs were performed without considering any time lag, because chlorophyll-*a* concentration presented a low cross-correlation (P>0.05) with both attributes within three months lag (S1 Fig, see Discussion). None of the models presented an auto-correlative temporal structure (P>0.05, within a seasonal time window; S2 Fig). The GLMs were followed by the likelihood ratio tests (analysis of deviance) available in the *MASS* package [64] in R. The seasonal probability of observing MSFFs and the seasonal probability that a certain species would engage in a MSFF, were calculated applying GLMs with a binomial error structure.

#### MSFF composition

In order to determine the seasonal variation in MSFF composition and relate its variations to the availability of food, multivariate generalized linear models (*multiGLM*) were performed with a negative binomial (total abundance) and binomial (presence/absence) error distributions, where seasonal composition was the dependent variables and seasonality with primary production were the predictive and interactive variables. The *multiGLM*s were followed by an analysis of deviance using the "PIT-trap" resampling with 10,000 iterations (which bootstraps probability integral transform residuals, which has shown the most reliable type I error rates [65]) and maximum likelihood test (likelihood ratio test). As a *post-hoc* for multiple comparisons between seasons, the *summary*.*glm* and *relevel* functions were used. In addition, univariate analyses (negative binomial error distribution) for each species with the same specifications as the multivariate analysis were done. The multivariate models did not show any pattern in the residual *vs* fits plot (hence no suggestion of failure of linearity and mean-variance assumptions; S3 Fig). Multivariate analyses were performed with the *mvabund* package [65,66] of R.

We excluded from statistical analysis those species that were seen in MSFFs only in one season and with a frequency of occurrence <5%, for considering them occasional in the area.

## Results

**Fig 2:**
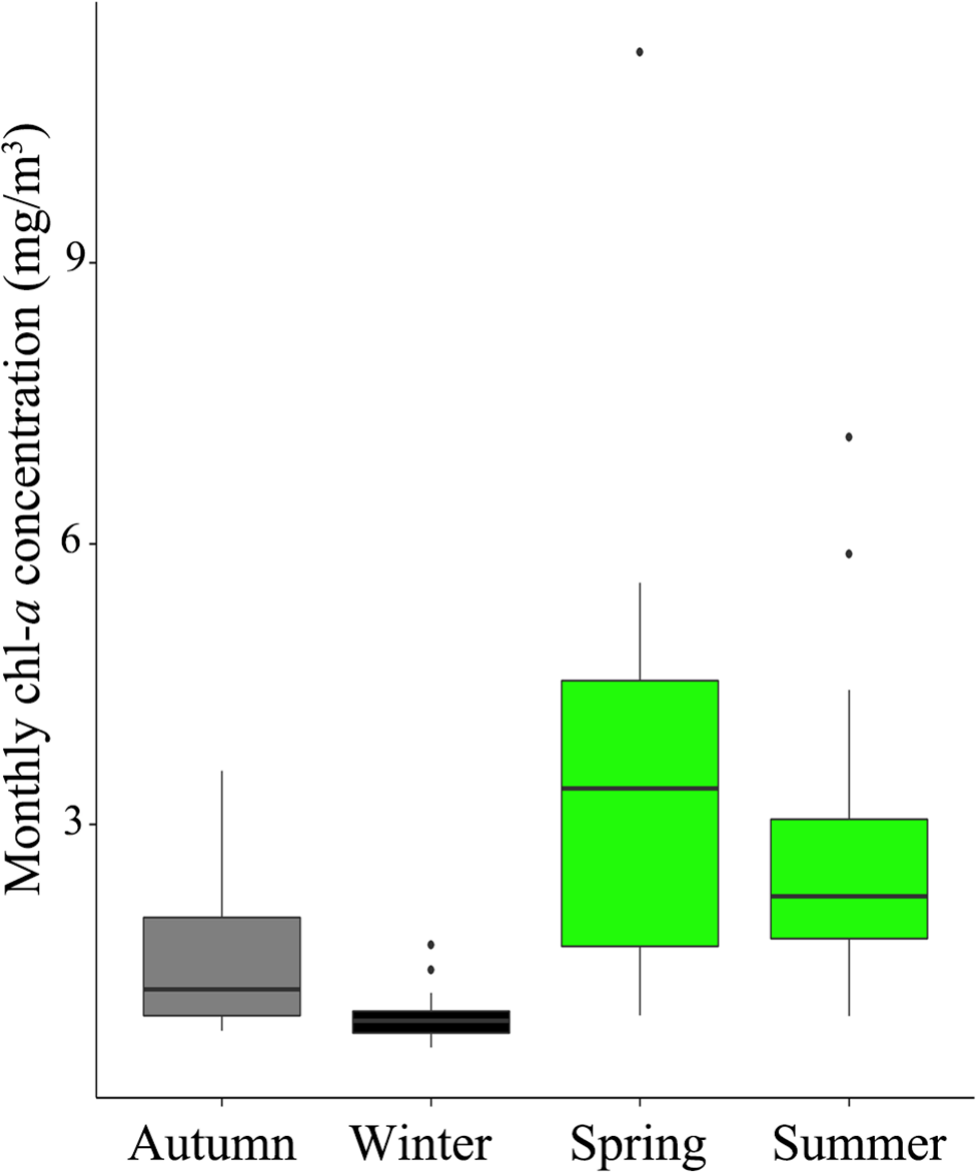
Box-plot of seasonal primary production (2006–2014) in the Valparaiso Bay. Different colors (green, gray and black) indicate significant differences between seasons (Mann-Whitney *U*; P<0.01). For considered area, see Fig 3. Chl-*a*: chlorophyll-*a* concentration.

**Fig 3:**
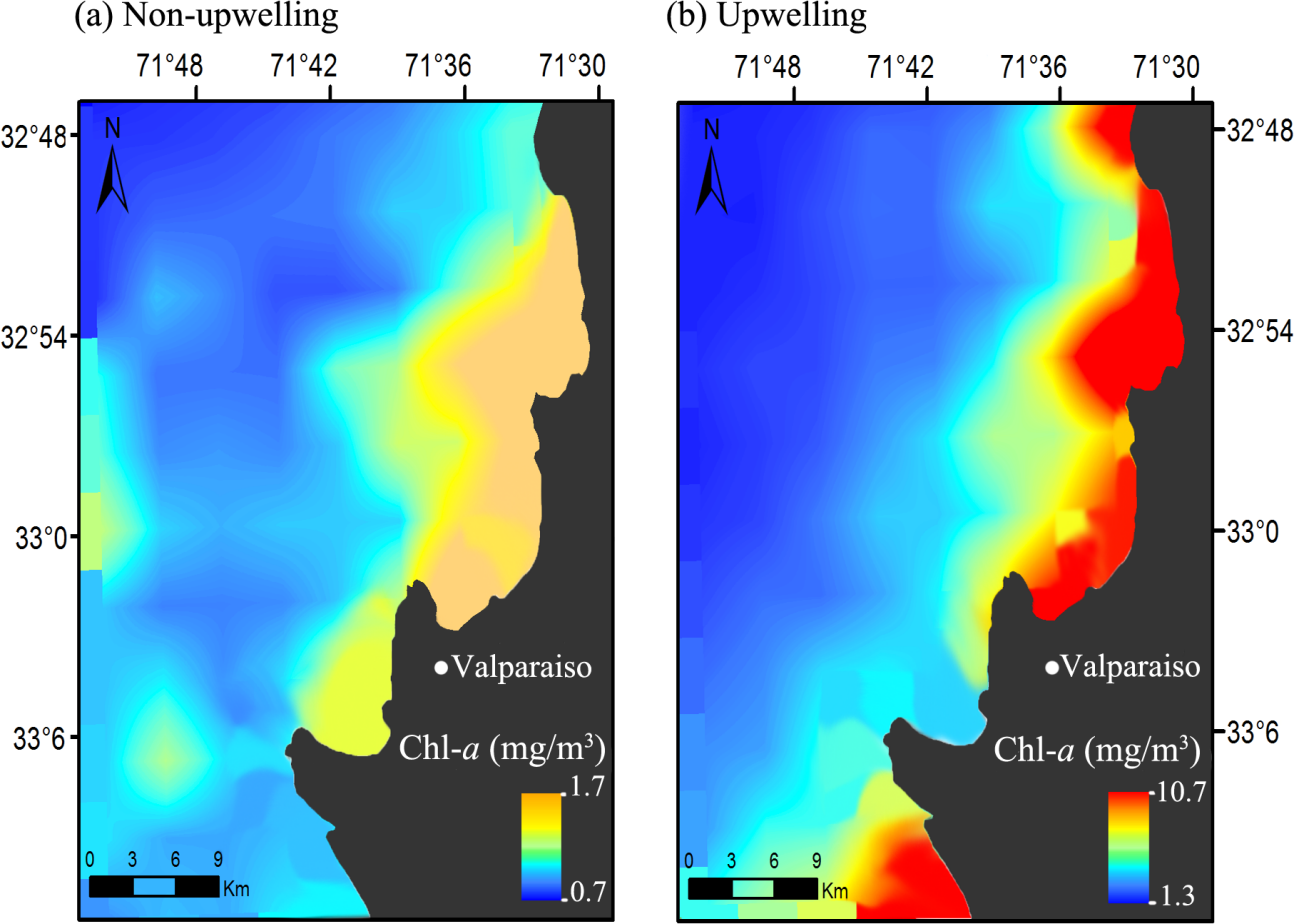
Spatiotemporal variation of primary production between the most contrasting seasons in the Valparaiso Bay. Average monthly concentration of chlorophyll*-a* (Chl-*a*); (a) winter (2006–2014) and (b) spring (2006–2014). This area was considered to extract mean monthly chlorophyll-*a* concentration values, which were used as predictors in GLMs.

Primary production at the coast of Valparaiso showed significant seasonal variation (Kruskal-Wallis *X* ^2^ = 47.48; P<0.001, see Fig 2 for *post hoc* comparisons) with spatiotemporal differences of up to one order of magnitude between spring and winter (Fig 3).

**Fig 4:**
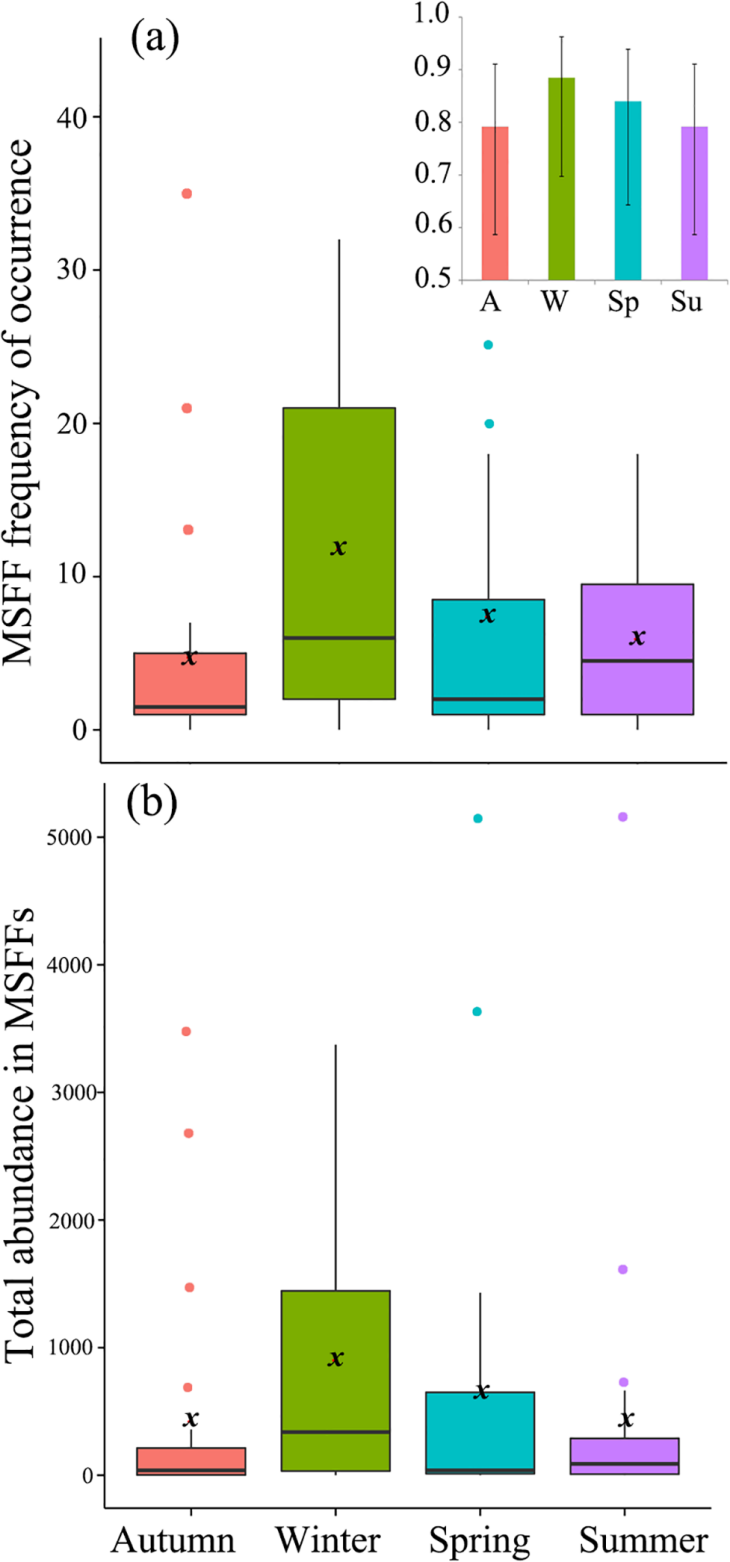
Box-plot of seasonal variation of MSFF attributes at Valparaiso Bay. (a) MSFF frequency of occurrence (MSFFs observed per month) and (b) MSFF abundance (total number of individuals observed in MSFFs per month). Insert (4a top) shows the monthly probability (± 95% confidence interval) of observing MSFFs per season (GLM, binomial structure). ***x*** shows the mean.

During the study period, we observed 38 species at Valparaiso Bay, of which 71% were engaged in MSFFs (Tables 1 and 2) and a total of 736 MSFFs; 16% in autumn, 41% in winter, 24% in spring and 20% in summer. The frequency of occurrence of MSFFs showed marginally significant seasonal differences (P = 0.053, Table 3A, Fig 4A), with the greatest frequency of occurrence in winter with an average of 12 MSFFs per month. In addition, winter presented the maximum probability of observing MSFFs per month (88%, Fig 4A top). Similarly, total abundance of individuals (birds per month) in MSFFs (Fig 4B) was highest in winter, but differences between seasons were not significant (P>0.05; Table 3B). Neither chlorophyll-*a* concentration nor its interaction with seasonality had a significant effect on the variation of both MSFF attributes (P>0.05; Table 3).

**Table 2:** Seasonal probability (95% confidence limits) that a certain species would engage in a MSFF at Valparaiso Bay.

	Autumn	(*L*-*U*)	Winter	(*L*-*U*)	Spring	(*L*-*U*)	Summer	(*L*-*U*)
**1**	SOSH	46–64	KEGU	58–69	KEGU	49–64	KEGU	51–66
**2**	KEGU	42–60	SOSH	39–50	SOSH	37–52	FRGU	36–52
**3**	GRGU	19–36	GRGU	27–37	GRGU	15–26	PEPE	18–32
**4**	FRGU	19–36	SOFU	15–24	SOFU	15–26	SOSH	12–25
**5**	DIPE	7–19	CAPE	12–20	PISH	12–23	INTE	6–16
**6**	BRGU	5–15	WHPE	9–17	CAPE	7–16	PISH	5–14
**7**	PEBO	4–14	BRGU	7–13	FRGU	4–12	PEBO	5–14
**8**	PISH	2–11	DIPE	5–12	MAPE	3–11	ARTE	5–14
**9**	PEPE	2–10	MAPE	4–9	INTE	3–9	DIPE	3–11
**10**	INTE	2–10	INTE	3–8	PEPE	2–9	GUCO	3–11
**11**	MAPE	2–10	PEPE	3–7	REPH	2–9	GRGU	1–8
**12**	BBAL	2–10	BBAL	1–5	WHPE	2–8	REPH	1–8
**13**	SPHE	2–10	GUCO	1–5	ARTE	2–7	PAJA	1–8
**14**	WHPE	1–9	PEBO	<0.5–4	BRGU	1–7	NECO	1–7
**15**	GUCO	1–9	HUPE	<0.5–3	GUCO	1–7	SAAL	<0.1–6
**16**	HUPE	1–9	SAAL	<0.1–4	BBAL	1–7	WIST	<0.1–6
**17**	NECO	1–8	SOTE	<0.1–4	SPHE	1–7	SPHE	<0.1–5
**18**	RECO	1–8	ARTE	<0.1–3	WIST	1–7	HUPE	<0.1–5
**19**	SOFU	<0.5–7	RECO	<0.1–3	SOTE	1–7	CHSK	<0.1–5
**20**	CHSK	<0.1–7	WEPE	<0.1–3	DIPE	1–6	RECO	<0.1–5
**21**	PAJA	<0.1–7	WIST	<0.1–3	PEBO	1–5	SOTE	<0.1–5
**22**	ARTE	<0.1–6	SPHE	<0.1–2	HUPE	1–5		
**23**	SAAL	<0.1–6	CHSK	<0.1–2	RECO	<0.5–4		
**24**	WEPE	<0.1–6			CHSK	<0.1–6		
**25**					SAAL	<0.1–4		
**26**					NECO	<0.1–4		
**27**					PAJA	<0.1–4		

Lower (*L*) and upper (*U*) 95% confidence intervals (as percentage) from binomial GLMs are shown. For species codes, see Table 1.

**Table 3:** Results of deviance analysis (GLM, negative binomial distribution) of frequency of occurrence and total abundance in MSFFs at Valparaiso Bay.

Attributes	Variable	Df	Deviance	Res.Df	Dev.Resi.	Pr(>Chi)
a) Occurrence	**NULL**	** **	** **	98	125.0	** **
	**Seasons**	3	7.6	95	118.0	**0.053**
	**Chl*-a***	1	1.3	94	116.7	0.255
	**Seasons x Chl*-a***	3	4.8	91	111.9	0.187
b) Abundance	**NULL**			98	134.8	
	**Seasons**	3	4.3	95	130.5	0.229
	**Chl*-a***	1	2.5	94	127.9	0.109
	**Seasons x Chl-a**	3	3.4	91	124.5	0.333

Df: degrees of freedom; Res.Df: residual degrees of freedom; Dev.Resi: residual deviance; Chl-*a*: chlorophyll-*a* concentration; x: indicates interaction between predictive variables. Null model contains only the intercept as a parameter.

**Fig 5:**
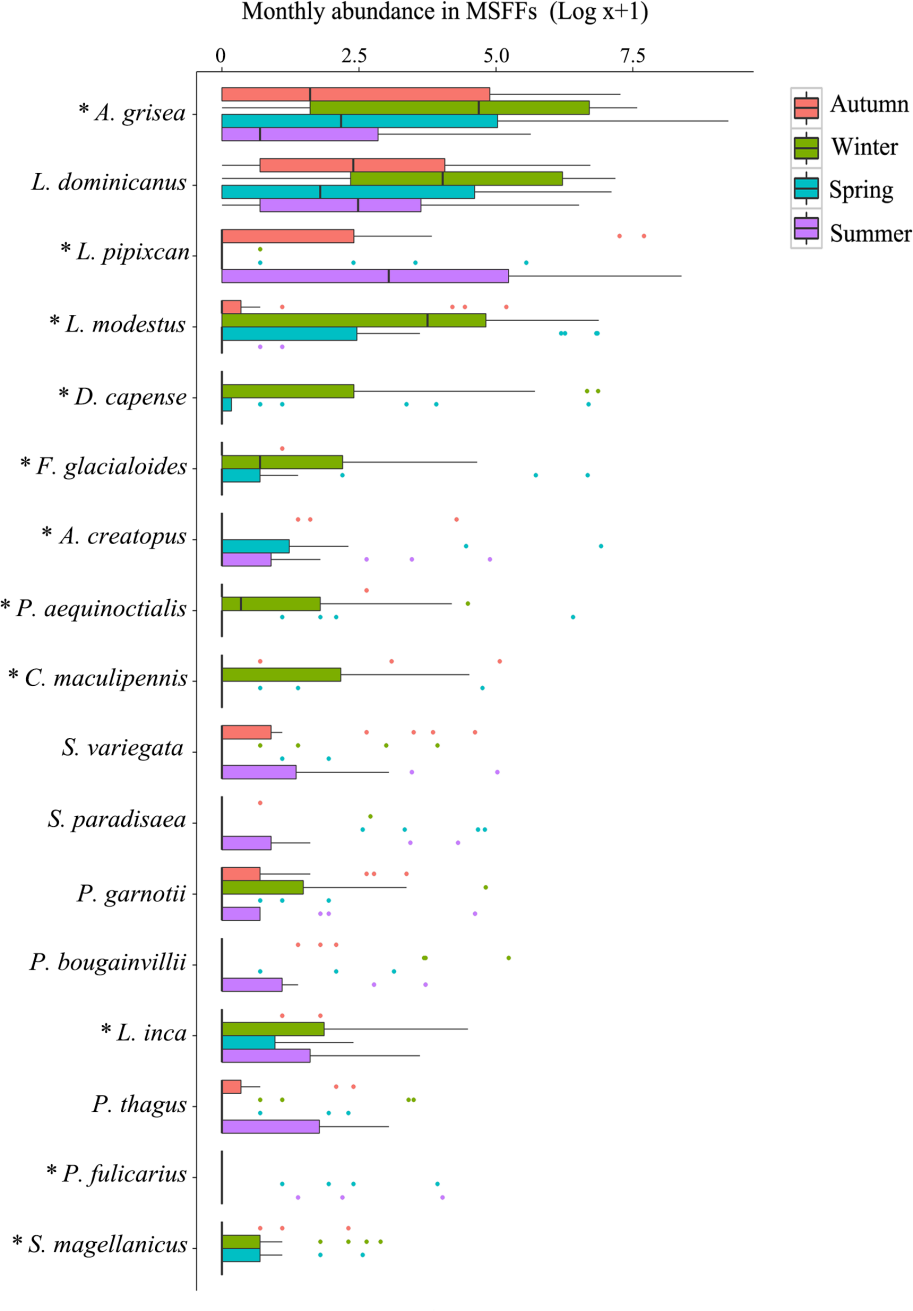
Box-plot of the seasonal variation in the abundance of each species involved in MSFFs (total number of individuals observed in MSFFs per month) at Valparaiso Bay. Species with frequency of occurrence ≥5% are displayed and sorted from highest to lowest abundance. * indicates species with significant seasonal variation (P<0.05, Table 1).

Multivariate analysis indicate that MSFF compositional variation (total abundance and presence/absence) is explained mostly by the seasonality (P<0.05; Table 4, Fig 5), with significant differences between all seasons (P<0.05; Table 5). Neither chlorophyll-*a* concentration nor its interaction with seasonality had a significant multiplicative effect on MSFF composition (P>0.05; Table 4). Species that showed significant seasonal variation are shown in Table 1 and Fig 5. For the vast majority of the abundant species in MSFFs at Valparaiso, there was a negative relation between monthly abundance and chlorophyll-*a* concentration (univariate analysis; Table 1), indicating that seabirds are engaged in MSFFs with higher abundances in months with lower primary production. However, this trend was significant (P<0.05) only for Southern fulmar, White-chinned petrel, Brown-hooded gull and Westland petrel (for scientific names, see Table 1). As in the multivariate analysis, the interaction of chlorophyll*-a* concentration and seasonality had no significant effect on any species (P>0.05, Table 1).

**Table 4:** Results of the deviance analysis of MSFF species composition (*multiGLM*) at Valparaiso Bay.

Model family	Variable	Res.Df	Df	Deviance	Pr(>Dev)
**a) Negative binomial**	**Seasons**	78	3	251.21	**<0.001**
	**Chl*-a***	77	1	48.35	0.090
	**Seasons x Chl*-a***	74	3	89.29	0.775
**b) Binomial**	**Seasons**	78	3	225.59	**<0.001**
	**Chl*-a***	77	1	33.95	0.410
	**Seasons x Chl*-a***	74	3	72.72	0.667

Models both with a negative binomial (total abundance) and binomial (presence/absence) error distribution are shown. Values obtained after 10,000 iterations using the resampling "PIT-trap". Res.Df: residual degrees of freedom; Df: degrees of freedom; Chl-*a*: chlorophyll-*a* concentration; x: indicates interaction between predictive variables.

**Table 5:** Multiple comparison of MSFF species composition between seasons at Valparaiso Bay.

	Autumn	Winter	Spring	Summer
**Autumn**	-	90.07	78.28	70.16
**Winter**	**0.008**	-	75.32	155.71
**Spring**	**0.016**	**0.015**	-	124.08
**Summer**	**0.005**	**0.001**	**0.002**	-

The model (negative binomial error distribution) was done considering only seasonality as a predictive variable. Numbers over the diagonal represent values of maximum likelihood (likelihood ratio) and bolded numbers are the respective p-value.

## Discussion

Based on studies that provide evidence that flocking is most valuable when food is both scarce and patchy [27–33], we predicted that in seasons with lower food availability (autumn-winter), MSFFs would be more frequent and abundant, while in seasons with greater food availability (spring-summer), MSFFs would be less frequent and would contain fewer individuals. Our results showed that Valparaiso Bay indeed presented a pronounced seasonality in primary production, with highest concentrations of chlorophyll*-a* in spring-summer and low concentrations of chlorophyll*-a* in autumn-winter seasons (with spatiotemporal differences up to an order of magnitude, see Thiel et al. [36] for a review). However, this marked seasonal variation in food availability had no significant influence on MSFF attributes, sparsely explaining their variations (frequency of occurrence, abundance and composition; P>0.05).

Although our results showed that MSFFs occur with highest frequency in winter (season with the lowest food availability), the probability of observing MSFFs was high for all seasons (≥80%) and total abundance did not show significant seasonal differences, demonstrating that this feeding strategy is used consistently and independently of seasonal food availability in this upwelling area. The negative relation between monthly abundance and chlorophyll-*a* concentration for most of abundant species at Valparaiso Bay is consistent with the foraging efficiency hypothesis, so it appears that during low resource availability, seabirds rely more on local enhancement to detect prey ([67], but not at a seasonal scale). However, the lack of significance for the vast majority of species is probably due to the complexity and variability of this phenomenon that responds to multiple variables besides food availability, such as density and functional groups of birds present in the area [8,9]. Our hypothesis thus is not supported by these results.

While spatial variation in food availability has been recognized as a key variable for composition and abundance in seabird MSFFs [20,24], temporal variation in food availability has received comparatively less attention and its effects on MSFF attributes have been poorly quantified. This is particularly true for eastern boundary current systems (e.g. Humboldt, Benguela and California) which are subjected to strong temporal variation in primary production [16–18,25]. Ainley and Boekelheide [68] found for the period 1971–1983 consistent interannual variations in the frequency of occurrence of MSFFs in the Farallon islands (California Current). These variations were attributed to food availability, as lower frequency of occurrence of MSFFs was evident when food availability was low. However, they noted that during these years, the behavior of some “catalytic” species (gulls) was more attractive for other species, mainly for Sooty shearwater (for scientific name, see Table 1), which were seen in numerous MSFFs [68]. These observations are consistent with our results, since winter MSFFs at Valparaiso Bay were composed mainly by gulls and Sooty shearwaters.

Remotely-sensed data (i.e. chlorophyll*-a*) are widely used as a proxy for inferring or predicting spatial and temporal at-sea distribution of meso-top predators. Notwithstanding, for seabirds its use has revealed mixed results [55–59], primarily based on the time lags between primary production and trophic levels higher up the food chain [69]. Gremillet et al. [56] showed that foraging seabirds (Cape gannet) match areas of high primary production at the Benguela Current, however, the same areas in turn strongly mismatch distribution of pelagic fish which was attributed to climate change and overfishing [56,70]. In the California Current primary production has shown strong potential predicting top-predators distribution patterns [57,58], with up to 90% of explained variation in the seabird density distributions [58].

Our study, although within a fine spatial scale, was focus on the seasonal variation in food availability. Studies in south-central Chile have found that the highest reproduction, abundance and biomass of the main zooplankton groups occur during spring and summer [36,38–42,71,72]. This suggests a strong synchronization between the timing of phyto- and zooplankton population growth, which could be even <2 weeks [73]. Furthermore, upwelling events itself can help concentrate zooplankton at coastal areas and in the upper ocean layer ([42,74] see Fig 3). These conditions, in turn determine planktivorous fish recruitment such as sardine (*Strangomera bentincki*) and anchovy, since these species concentrate their spawning in late winter [75,76], prior to the increased planktonic abundance promoted by coastal upwelling during spring [75]. Gomez et al. [77] found a high correlation (0.92) between spring chlorophyll-*a* and common sardine recruitment, suggesting that strong upwelling conditions during spring increases pre-recruit survival, whereas the opposite occurs with weak upwelling conditions. Similar results were recently shown by Silva et al. [78] for common sardine relative abundance, which is consistent with the evolutionary hypothesis that planktivorous fishes at HCS have adapted their spawning period in response to seasonal oceanographic conditions, in order to match maximal food concentration [75,79]. Considering this evidence of reduced time lag between primary producers and trophic levels higher up the food chain, particularly at HCS, we validate the use of chlorophyll-*a* concentrations as a good proxy for seasonal seabird food availability in central Chile.

The observed pattern for MSFF attributes at Valparaiso Bay, rather than seasonal food availability, suggests an important influence of reproductive and migratory (boreal and austral migrants) habits of birds (see [80]) which modulate MSFF dynamics throughout the whole year. The highest frequency of occurrence of MSFFs observed in winter is probably a result of an enhanced bird density at Valparaiso Bay through the influx of migrants. Central Chile is characterized by high richness and abundance of resident, endemic and migratory seabirds that use this area as a commuting, stopover and wintering areas within their migratory flyway along the southeastern Pacific [43–46,81]. Austral migratory species (e.g. Cape petrel, Southern fulmar, White-chinned petrel and Sooty shearwater) showed the greatest frequency of occurrence and abundance in the MSFFs during winter at Valparaiso Bay. The Sooty shearwater breeds in New Zealand, Australia, Chile and the Falkland Islands during the austral summer [82] and perform extensive trans-equatorial post-breeding migrations (in austral autumn) within the Pacific Ocean to their wintering grounds in western, central or eastern North Pacific [83]. Their highest abundance seen in MSFFs in winter at Valparaiso Bay suggest that this species overwinters at these latitudes and widely used this feeding strategy during this season, but also during their northward post-breeding migrations (austral autumn) and during their southward migration back to their breeding grounds (austral spring).

Sooty shearwater had the lowest occurrence and abundance in MSFFs during summer, as this species was likely concentrated at their breeding grounds during this time. A similar pattern to that of Valparaiso Bay has been observed for this species in their main wintering areas in the northern hemisphere [8,18]. Therefore, our observations adhere to existing evidence that Sooty shearwater is a key species on structuring seabird assemblages, probably throughout all its distributional range [8,44]. Cape petrel, Southern fulmar and White-chinned petrel breed in Antarctica and subantarctic islands and have a post-breeding migration to their wintering areas into both Atlantic and Pacific Oceans within the southern hemisphere [81,82,84]. All three species showed a marked change in relation to seasonality with greatest abundances in MSFFs during winter. In northern Chile, Weichler et al. [25] demonstrated that the occurrence of MSFFs had a profound effect on the distribution of HCS endemic seabirds, with up to 95% of occurrence for Gray gull. Among boreal trans-equatorial migration species, Franklin’s gull was the most abundant species at Valparaiso Bay. This species breed in the United States and Canada and migrates along the coast of the Pacific Ocean to overwinter at the HCS, where it has been seen in foraging flocks with up to 500 birds [85]. Their high abundances in summer suggest that MSFFs are important part of their feeding strategies during their overwinter (austral summer). Finally, the most abundant species in all seasons was the Kelp gull. This relatively sedentary species is widely distributed in the southern hemisphere; at Chilean coasts, it is the most common gull and breeds along the entire coast [86]. In northern Chile, their distribution and abundance has been associated mainly with anthropogenic sources of food supply [25,87,88]. In contrast, Nasca et al. [23] found that this species was one of the most abundant in MSFFs in Argentina, probably acting as a “catalyst”. This, in conjunction to our results, demonstrates the versatility and plasticity of Kelp gull to obtain their food, which is certainly associated with its wide distribution and current population trends [89,90].

All migratory and resident species mentioned above showed a significant influence on the composition and temporal dynamics of the MSFFs at Valparaiso Bay, probably providing and using “social cues” [2] that would allow them to reduce resources unpredictability. Indeed, the Sooty shearwater has been described as a “joiner/suppressor” and “catalyst” species of MSFFs in northern hemisphere [8,18,68] and similarly, the “catalyst” role has been well documented for gulls [8,18,22,26]. Additionally, gulls (Kelp, Gray and Franklin’s gulls) and Sooty shearwater were the most abundant species in the MSFFs at Valparaiso Bay, which is consistent with the density-dependence of local enhancement [9,14]; at higher flock density, more birds will be more effective in locating food patches up to a threshold [14]. Therefore, either as “catalyst” or “joiners/suppressor”, these species certainly play a major role in the dynamics of MSFFs at Valparaiso Bay through local enhancement.

In conclusion, our study demonstrates that the contrasting seasonal variation in food availability of the study area did not show any significant relation with MSFF attributes, in fact the high probability of observing a MSFFs in all seasons suggests that this feeding strategy is commonly used by birds and occur independently of seasonal food availability, allowing them to reduce resources unpredictability throughout the whole year in this highly variable environment (at least at the spatial scale considered in our study). In addition, our results show that MSFF dynamics are modulated mainly by bird’s phenology with a strong influence of boreal and austral migratory species, which undoubtedly play a key role in seabird assemblages at Valparaiso Bay. Our study adheres to the growing evidence that local enhancement in seabirds is a relevant foraging strategy at an ecological time scale with deep evolutionary implications [5,11–14,91].

## Supporting Information

S1 Fig Cross-correlation plot between primary production and MSFF attributes. (a) MSFF frequency of occurrence and (b) MSFF total abundance. Note that there is a low cross-correlation (P>0.05) within three months lag.
(TIF)

S2 Fig Auto-correlation plot for the residuals of GLMs. (a) MSFF frequency of occurrence and (b) MSFF total abundance. Note that there is a low auto-correlation (P>0.05) within four months lag. In addition, Durbin-Watson test of model residuals are shown.
(TIF)

S3 Fig Residual *vs* fits plot to check assumptions of *multiGLM*s (with different species coded in different colors). (a) Negative Binomial and (b) binomial error distributions.
(TIF)

S1 Table Seasonal data on MSFF frequency of occurrence and abundance and values for primary production. (XLSX)

S2 Table Seasonal data on species abundance and values for primary production. (XLSX)
