# Supplementary figures and images for: Influence of Seasonal Food Availability on the Dynamics of Seabird Feeding Flocks at a Coastal Upwelling Area
Source: PLoS One. 2015 Jun 30;10(6):e0131327. doi: 10.1371/journal.pone.0131327 (PMC4488391; doi:10.1371/journal.pone.0131327)

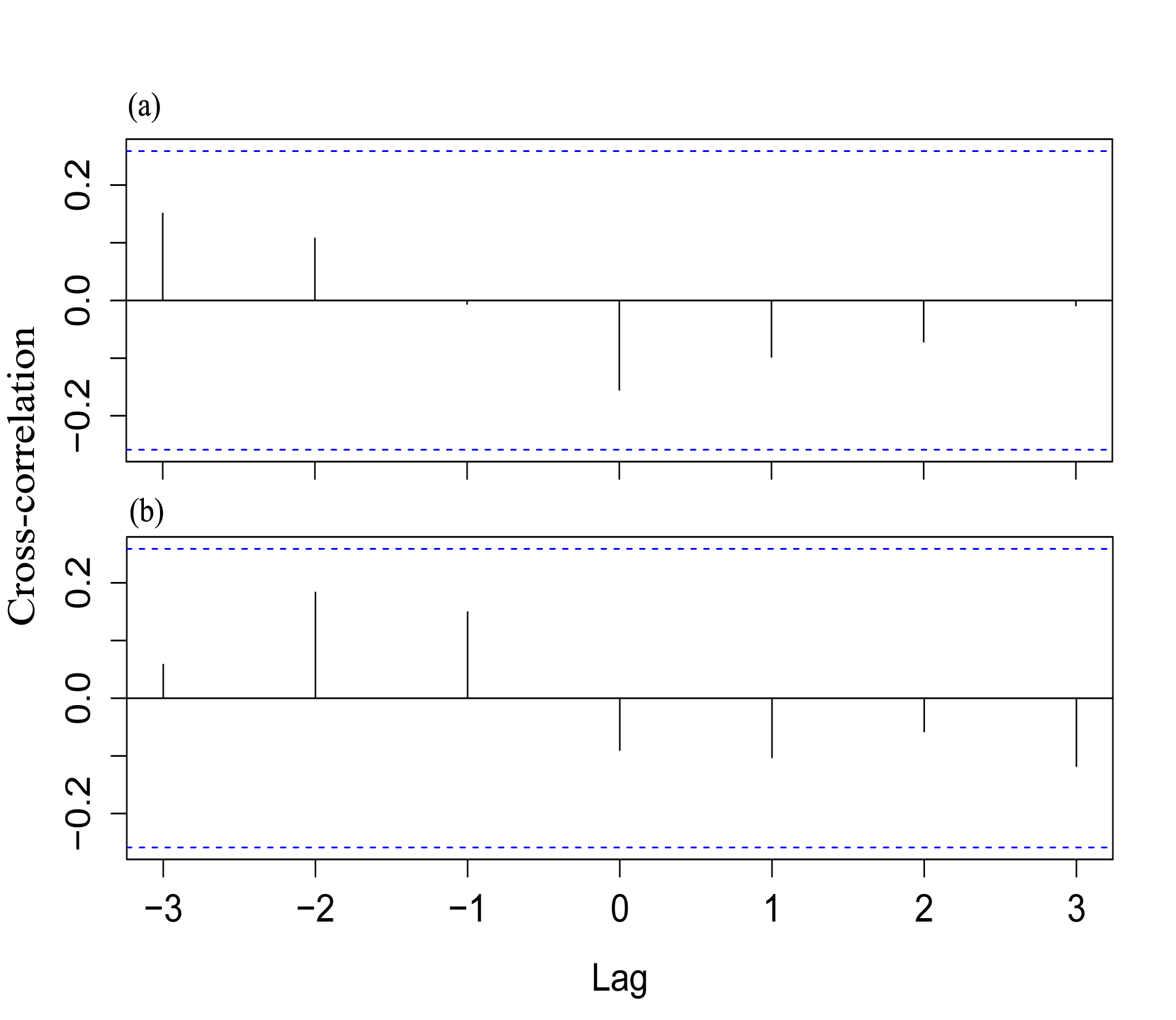

Supplement: S1 Fig — (a) MSFF frequency of occurrence and (b) MSFF total abundance. Note that there is a low cross-correlation (P>0.05) within three months lag. (TIF) [file pone.0131327.s001.tif]

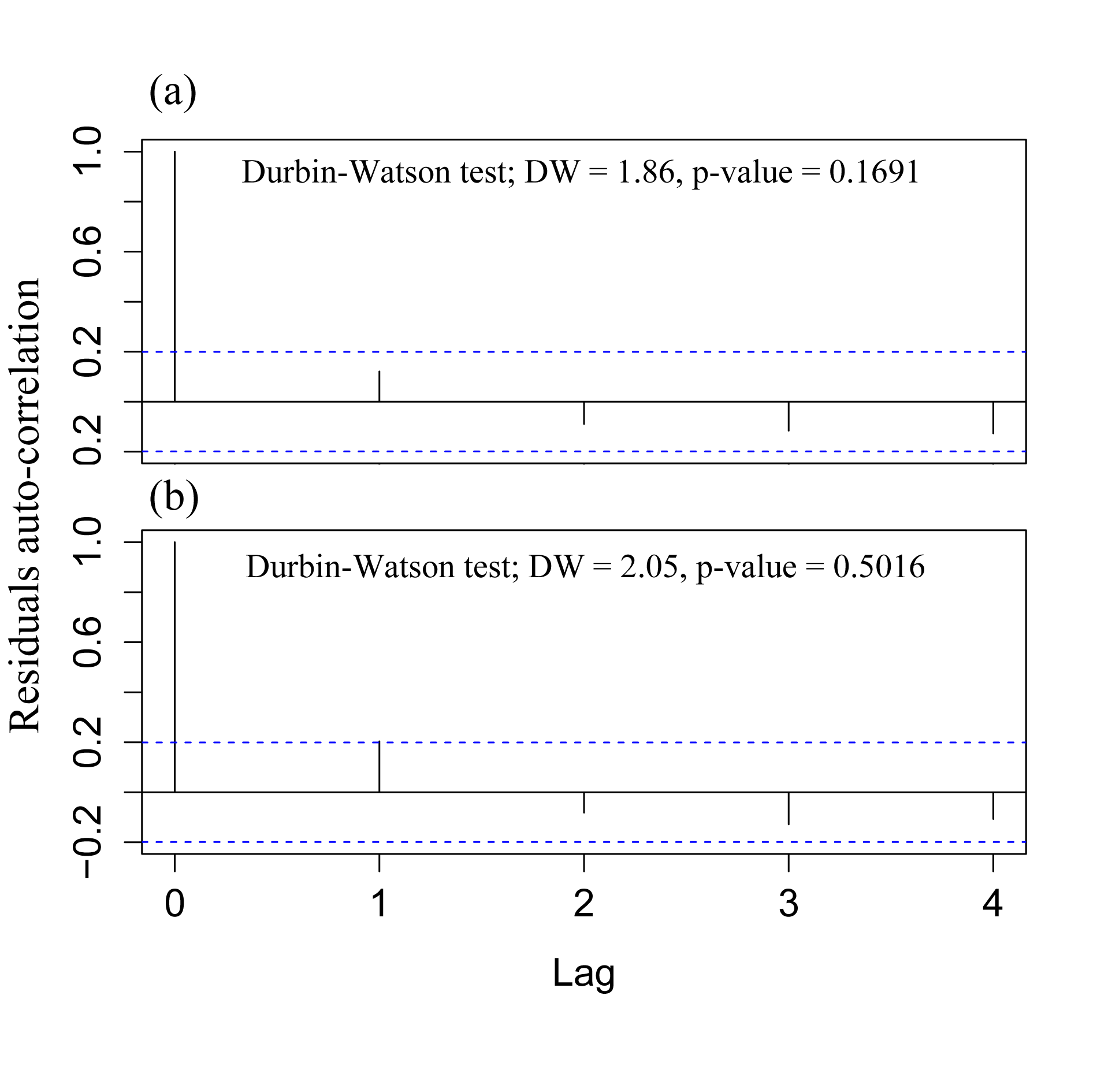

Supplement: S2 Fig — (a) MSFF frequency of occurrence and (b) MSFF total abundance. Note that there is a low auto-correlation (P>0.05) within four months lag. In addition, Durbin-Watson test of model residuals are shown. (TIF) [file pone.0131327.s002.tif]

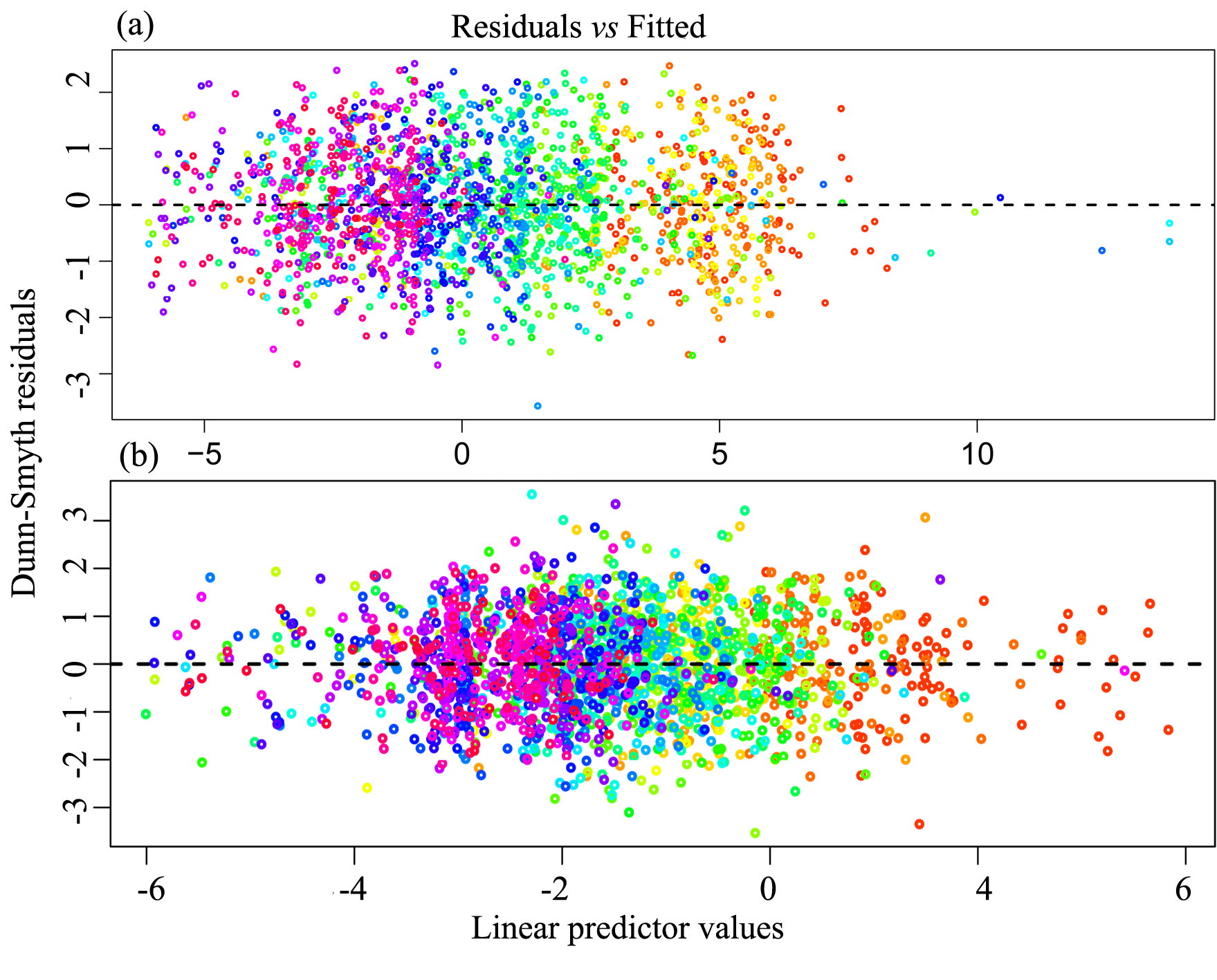

Supplement: S3 Fig — (a) Negative Binomial and (b) binomial error distributions. (TIF) [file pone.0131327.s003.tif]
